# The role of child BMI growth in neurodevelopment and school readiness—Current landscape and future directions

**DOI:** 10.1111/ppe.13132

**Published:** 2024-10-04

**Authors:** Yi Ying Ong

**Affiliations:** ^1^ Department of Paediatrics, Yong Loo Lin School of Medicine National University of Singapore Singapore City Singapore

In a review published almost two decades ago, Taras and Potts‐Datema^1^ found consistent associations between child obesity and school performance, despite the limited literature available then. Since then, a growing body of literature suggests that child obesity is associated with poorer neurocognition and executive function.^2^ Emerging research sheds light on several potential biological mechanisms mediating these associations, such as neuroinflammation and changes in brain structure and function.^2^ However, in a more recent systematic review, Santana et al.^3^ found no strong evidence supporting the link between child obesity and school performance. As aptly summarised by Santana et al., the lack of consistent associations might be due to gaps in the field, which include (i) the predominance of cross‐sectional studies investigating body mass index (BMI) at a single time point, (ii) the lack of adjustment for confounders and (iii) inadequate sample sizes.^3^

In this issue of *Paediatric and Perinatal Epidemiology*, Li and colleagues^4^ addressed these gaps by investigating the associations of longitudinal BMI growth (0–4 years) with school readiness assessed at kindergarten (4–6 years), adjusting for potential confounders, including socioeconomic status and maternal education. The authors conducted the study in a relatively large prospective cohort of 1077 healthy children enrolled in the TARGet Kids! research network in Canada. School readiness, measured by the early development instrument, assessed five developmental domains: (i) physical health and well‐being, (ii) social competence, (iii) emotional maturity, (iv) language and cognitive development, and (v) communication skills and general knowledge. ‘Overall vulnerability’ in school readiness is defined as having a score below the 10th percentile cut‐off for their corresponding kindergarten grade level in at least one of the five domains.

Using two different analytical approaches, Li et al. found that BMI z‐score (z‐BMI) growth rates (0–2 years, 2–4 years) and z‐BMI catch‐up trajectory (vs. stable trajectory) were not associated with overall vulnerability in school readiness. The authors suggested that, unlike overweight/obesity status, BMI growth might not capture more severe levels of obesity, and hence, there might be small effects that were not reflected.

It is commendable that the authors utilised two analytical approaches for examining longitudinal BMI trajectories from ages 0 to 4 years: Piecewise linear mixed effects modelling and latent class mixed modelling. The former approach quantifies period‐specific BMI growth rates (0–2 years; 2–4 years), while the latter allows for identifying distinct overall BMI growth trajectories from 0 to 4 years. These two approaches complement and shed additional insights on the relationship between BMI growth and school readiness outcomes.

Another strength of the study was the investigation of effect modification by sex. While the authors did not find effect modification, it is good practice to investigate relevant effect modifiers/interactions to gain a deeper understanding of the relationship between exposure and outcome.

I highlight three limitations that are relevant to consider when interpreting the findings. First, school readiness was measured by teacher‐reported school readiness based on the early development instrument. As the authors explained, this measure, while validated, might not be sensitive enough to capture differences in neurocognition compared to objective test scores or magnetic resonance imaging (MRI) measures of neurodevelopment. Teacher‐reported school readiness is subject to biases in teachers' perceptions. For instance, increasing z‐BMI from the fifth to eighth grade has been associated with worsening teacher perceptions of academic ability, regardless of objectively measured ability from standardised test scores.^5^

Second, in latent class mixed modelling, the authors chose the two‐trajectory model based on the lowest Bayesian information criterion. However, the two trajectories had relatively high overlap, making it difficult to identify children with an ‘at‐risk’ growth trajectory compared to a ‘normal’ growth trajectory. As many as 41.5% of children were in the catch‐up trajectory, while 58.5% were in the stable reference trajectory. This stands in contrast to other population‐based cohort studies which have identified more than two distinct trajectories of BMI z‐score growth among children. For instance, a recent study has identified five distinct z‐BMI trajectories (stable normal low, stable normal, stable normal high, early acceleration, late acceleration) from birth to childhood, with the early acceleration (5.8%) and late acceleration (8.4%) trajectories likely representing the ‘at‐risk’ trajectories as they were associated with elevated cardiometabolic risk markers at age 6 years.^6^ As the authors conceded, perhaps in the TARGet Kids! Cohort, there might not be enough variation to distinguish distinct subgroups clearly. The overlapping trajectories might dilute the strength of associations between the growth trajectories identified (catch‐up trajectory vs. reference) and school readiness.

Third, while the authors adjusted for a reasonable list of important confounders, including socioeconomic factors and child characteristics, the associations might still be biased by unmeasured confounders. Additional relevant confounders include the father's education, parity, parents' ethnicity and parents' BMI. These factors might affect parenting practices and the home environment, affecting child BMI growth and school readiness.

While Li et al. concluded that there were no associations, the consistency in the direction of results across different analytical approaches should be considered in light of existing literature. Specifically, Li et al. consistently reported positive directions of association between increased BMI growth and increased overall vulnerability in school readiness—whether for BMI z‐score growth rates from 0 to 2 years (risk ratio [RR], 1.10, 95% confidence interval [CI] 0.78, 1.55) or being in the BMI z‐score catch‐up trajectory (vs. stable trajectory) (RR, 1.05, 95% CI 0.82, 1.35). Notably, these findings were consistent with an earlier study conducted by the authors in the same cohort of children enrolled in TARGet Kids!, where child overweight/obesity (vs. normal weight/underweight) was associated with poorer school readiness (odds ratio [OR] 1.60, 95% CI 1.02, 2.50).^7^ In the earlier study, the authors also investigated BMI z‐score as a continuous variable in place of child overweight/obesity. They reported a positive direction of association with overall vulnerability in school readiness (OR, 1.16, 95% CI 0.96, 1.39).^7^ Taken together, these findings from TARGet Kids! suggest small positive associations between child BMI/BMI growth and overall vulnerability in school readiness, especially in children with overweight/obesity.

These findings from TARGet Kids! are unsurprising in the context of existing literature. In 2020, Segal et al. systematically reviewed 22 studies from high‐income countries that used robust causal inference approaches. They found that childhood overweight/obesity was associated with poorer educational outcomes, with stronger effects observed at ages 12 and older.^8^

Two other recent studies in 2023 largely supported these findings. The first was a study of 4936 Grade 4 students in the United States, which found a weak association between weight status and academic achievement, with stronger associations among children who developed overweight/obesity.^9^ The other was a study of 1052 children in the United States that found small associations between BMI growth rate and cognitive test scores, with effect estimates ranging from 0.2 to 1.2 points lower standardised cognition test scores per standard deviation (SD) increase in BMI growth rates.^10^ All these emerging findings are concerning, implying that the rising childhood obesity epidemic might impact educational outcomes and human capital development.

I propose several avenues for enhancing future research:

- Longitudinal BMI measurements should be used to identify critical growth periods and explore potential sensitive phases of growth related to neurodevelopment. This would enable targeted monitoring and interventions.
- Since BMI serves as a convenient proxy for adiposity, it does not distinguish between fat and lean mass. With advancing technology, future research should assess total body fat and organ‐specific fat accumulations (e.g. abdominal, liver, intramyocellular) using advanced techniques such as MRI, quantitative magnetic resonance (QMR) and magnetic resonance spectroscopy (MRS).
- Objective neurodevelopment assessments, including standardised cognitive and developmental assessments administered directly to children, and evaluations of brain structures and functional connectivity, should be prioritised to minimise bias.
- The potential interaction between fetal and postnatal growth on neurodevelopment should be explored. Prior research suggests that postnatal ‘catch‐up growth’ may be particularly beneficial for children who experience poor fetal growth.^10^ Therefore, considering fetal and postnatal growth together may yield more accurate insights into neurodevelopmental outcomes.

Further investigation can clarify how child obesity phenotypes, growth trajectories and fat accumulation impact neurodevelopment and school readiness, potentially informing targeted interventions to support future school performance and human capital.

## ABOUT THE AUTHOR

**Yi Ying Ong** is a Research Assistant Professor in the Department of Paediatrics at the National University of Singapore. She completed her PhD in epidemiology from the National University of Singapore and post‐doctoral training at the Harvard TH Chan School of Public Health. She has worked closely with the Growing Up in Singapore Towards Healthy Outcomes (GUSTO) and Project Viva cohorts, focusing on early life growth, cardiometabolic health, and neurodevelopment.

## AUTHOR CONTRIBUTIONS

Ong is solely responsible for the contents of this commentary.

## CONFLICT OF INTEREST STATEMENT

The author declares no conflicts of interest.

## Data Availability

None.
